# Redundant Functions for Nap1 and Chz1 in H2A.Z Deposition

**DOI:** 10.1038/s41598-017-11003-8

**Published:** 2017-09-07

**Authors:** Raghuvar Dronamraju, Srinivas Ramachandran, Deepak K. Jha, Alexander T. Adams, Julia V. DiFiore, Michael A. Parra, Nikolay V. Dokholyan, Brian D. Strahl

**Affiliations:** 1 Department of Biochemistry and Biophysics, University of North Carolina at Chapel Hill, Chapel Hill, NC 27599 USA; 2 Curriculum in Genetics and Molecular Biology, University of North Carolina, Chapel Hill, NC 27599 USA; 3 Program in Molecular and Cellular Biophysics, University of North Carolina at Chapel Hill, Chapel Hill, NC 27599 USA; 4 Lineberger Comprehensive Cancer Center, University of North Carolina at Chapel Hill, Chapel Hill, NC 27599 USA; 5 Present Address: Division of Basic Sciences, Fred Hutchinson Cancer Research Center, Seattle, WA 98109 USA; 6 Present Address: Division of Hematology/Oncology, Department of Medicine, Children’s Hospital Boston, Boston, MA USA; 7 Present Address: Department Susquehanna University, Selinsgrove, PA 17870 USA

## Abstract

H2A.Z is a histone H2A variant that contributes to transcriptional regulation, DNA damage response and limits heterochromatin spreading. In *Saccharomyces cerevisiae*, H2A.Z is deposited by the SWR-C complex, which relies on several histone chaperones including Nap1 and Chz1 to deliver H2A.Z-H2B dimers to SWR-C. However, the mechanisms by which Nap1 and Chz1 cooperate to bind H2A.Z and their contribution to H2A.Z deposition in chromatin is not well understood. Using structural modeling and molecular dynamics simulations, we identify a series of H2A.Z residues that form a chaperone-specific binding surface. Mutation of these residues revealed different surface requirements for Nap1 and Chz1 interaction with H2A.Z. Consistent with this result, we found that loss of Nap1 or Chz1 individually resulted in mild defects in H2A.Z deposition, but that deletion of both Nap1 and Chz1 resulted in a significant reduction of H2A.Z deposition at promoters and led to heterochromatin spreading. Together, our findings reveal unique H2A.Z surface dependences for Nap1 and Chz1 and a redundant role for these chaperones in H2A.Z deposition.

## Introduction

Eukaryotic chromatin is regulated by multiple mechanisms that include post-translational histone modifications, ATP-dependent chromatin remodeling, and replacement of canonical histones with histone variants^1^. While the canonical histones are primarily deposited in the S-phase of the cell cycle, histone variants are synthesized in a replication-independent manner with their deposition and eviction at specific genomic loci maintaining distinct chromatin states^1, 2^. Variants of histone H2A are the most common and are found in most organisms from yeast to humans. H2A.Z (Htz1 in budding yeast) is a histone H2A variant that is highly conserved across species^2–4^. H2A.Z has been shown to be crucial for a variety of processes involving transcriptional activation and/or repression^5–7^, maintenance of heterochromatin^8^ and DNA damage response^9, 10^.

The function of H2A.Z is, in part, modulated by regulated deposition and eviction from chromatin^4, 9^. The major factor for regulated deposition of H2A.Z in chromatin in budding yeast is the SWR1 complex (SWR-C)^11^. SWR-C is an ATP-dependent chromatin-remodeling complex that recognizes the acidic surface on the H2A.Z-H2B dimer^12^ to deposit it into chromatin. Additionally, histone chaperones Nap1 and Chz1 have been implicated to work in close association with SWR1 complex in the deposition of H2A.Z into chromatin^4^. Nap1 is involved in the import of H2A.Z into the nucleus and Nap1 together with Chz1 delivers H2A.Z to Swr1-deposition machinery^13^. However, removal of Chz1 and/or Nap1 does not severely impact H2A.Z levels globally as detected by immunoblotting of the whole cell lysates^14^. In this study, we characterize the differential interactions between H2A.Z and its cognate chaperones – Nap1 and Chz1. Using structural modeling and discrete molecular dynamic (DMD) simulations, we discovered specific residues of H2A.Z that are critical for interactions with either Chz1 or Nap1. Mutations in H2A.Z and/or deletion of both Nap1 and Chz1 lead to severe biological consequences such as decreased survival in the presence of genotoxic or transcriptional stressors and defects in H2A.Z deposition that result in heterochromatin spreading.

## Results

### Constraint-driven Chz1-H2A.Z-H2B (CZB) structural ensemble generated by Discrete Molecular Dynamics simulations

Nap1 and Chz1 each contribute to H2A.Z deposition through loading H2A.Z-H2B dimers onto SWR-C. While the structure of Chz1 in complex with H2A.Z has been determined using NMR, no structures of Nap1 in complex with H2A.Z currently exists. The NMR and biochemical data suggest Chz1 to be an intrinsically unstructured protein that does not adopt a compact globular fold or significant secondary structure even upon binding to H2A.Z-H2B dimer^15^. Hence, to better understand the molecular recognition of H2A.Z-H2B by Chz1, we performed replica-exchange Discrete Molecular Dynamics (DMD) simulations to sample multiple conformations of the Chz1-H2A.Z-H2B complex (CZB) (simulation details in Methods). To ensure that the set of conformations (ensemble) of CZB we used for structural analysis reflected native conformations, we used a set of filters to select a subset of structures from all the snapshots obtained from the DMD simulations. Using filters that included potential energy, electrostatics and violations of published Nuclear Overhauser Effect (NOE) spectra of the CZB complex^15^ we arrived at an ensemble of structures for the CZB complex (the DMD ensemble). In our DMD ensemble, the average violation of NOE constraints defining the interface between Chz1 and H2A.Z-H2B is 0.04 Å, lower than the average violation of interface NOE constraints in the published NMR ensemble (0.07 Å), thus indicating that the DMD ensemble of the CZB complex has excellent agreement with experimental distance restraints, while at the same time extensively sampling interactions between Chz1 and H2A.Z-H2B.

### Diverse interactions drive specific recognition of H2A.Z-H2B by Chz1

**Figure 1:**
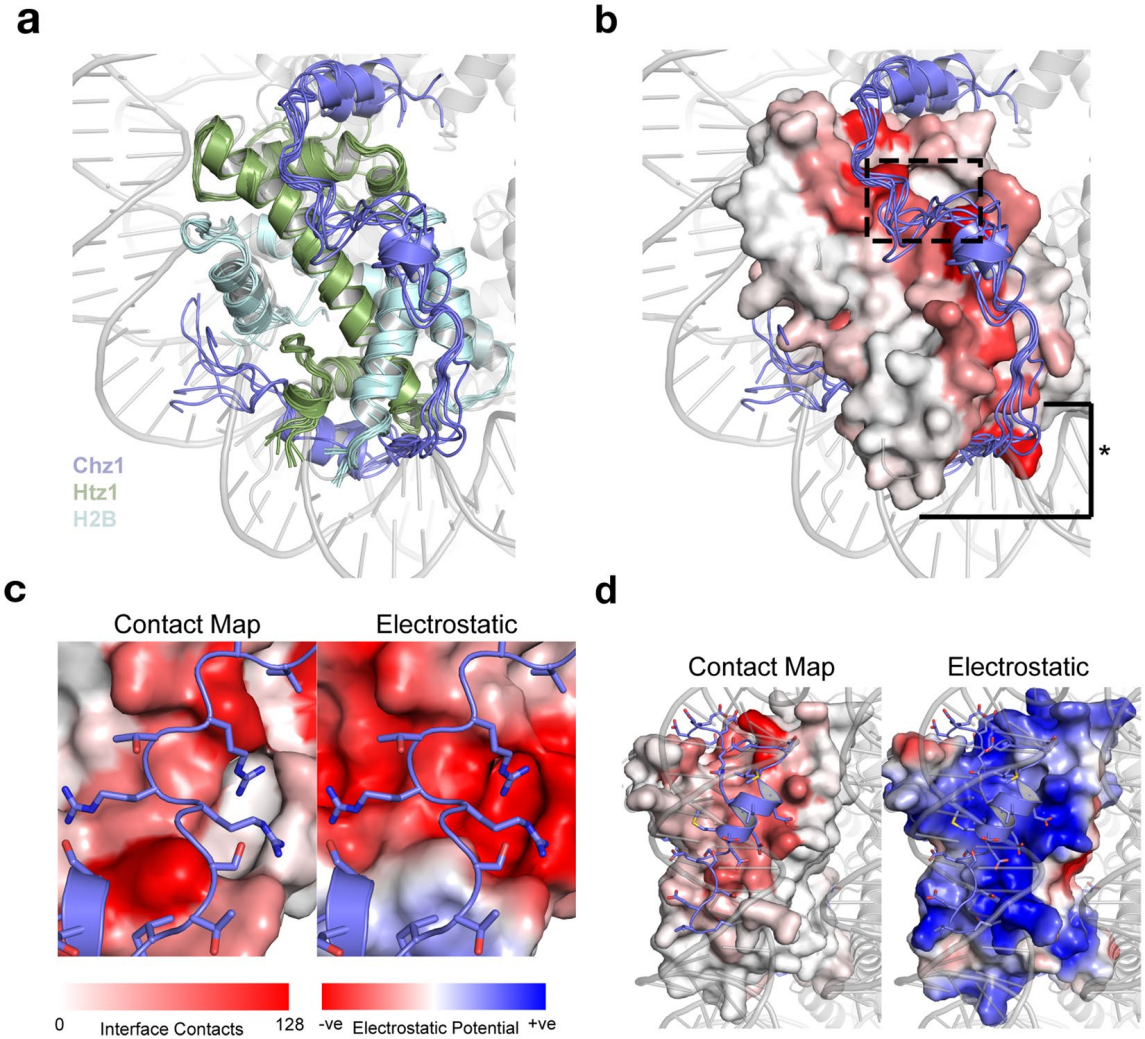
Molecular recognition and structure of H2A.Z-H2B by Chz1. (**a**) The six centroid structures from the refined DMD ensemble of H2A.Z-H2B-Chz1 complex are structurally aligned and displayed using cartoon representation. The centroid structures are overlaid over the yeast nucleosome structure in gray, highlighting the surface on H2A.Z-H2B that is bound by both Chz1 and DNA. (**b**) The H2A.Z-H2B dimer is displayed using surface representation with the residues colored according to the average number of interface contacts they form with Chz1, which is displayed with the cartoon representation. The structures are overlaid over the yeast nucleosome structure in gray. The dashed-box indicates the region of H2A.Z-H2B surface that forms the acidic patch. The asterisk indicates the DNA-binding surface of H2A.Z-H2B. (**c**) The acidic patch from the H2A.Z-H2B dimer is shown using surface representation forming interactions from three arginine residues from Chz1. (**d**) The DNA binding surface of H2A.Z-H2B is shown colored according to interface contacts. The nucleosome structure in gray is overlaid to highlight the path of DNA on this surface.

The histone recognition domain of Chz1 displays no secondary structure compared to globular, well-folded histone-recognition domains of many histone chaperones whose structures are known (e.g., Asf1^16, 17^, Nap1^18^, Rtt106^19^ and DAXX^20, 21^). However, the extended coil structure of Chz1 enables an extensive and specific interface with H2A.Z-H2B. The histone recognition motif forms a lasso-like structure, covering two thirds of the circumference of H2A.Z-H2B (Fig. 1a) and the chaperone-histone interface buries 2462.2 ± 151.5 Å^2^ solvent accessible surface area on average. To determine the binding interface of Chz1, we calculated average number of heavy-atom contacts formed by each residue of H2A.Z-H2B with residues of Chz1 in the DMD ensemble. When we represent this binding interface as a heat map on the H2A.Z-H2B surface (Fig. 1b), we observe specific regions on the surface of H2A.Z-H2B that form interactions with Chz1. The highly negatively charged “acidic patch” of H2A.Z-H2B is specifically bound by a series of three arginine residues in Chz1 (R105, R106, R108, Fig. 1c). The DNA-binding surface of H2A.Z-H2B, which is highly positively charged, is bound by a negatively charged, highly complementary surface of Chz1 (Fig. 1d).

### Interrogating the H2A.Z-H2B binding surface of Chz1

**Figure 2:**
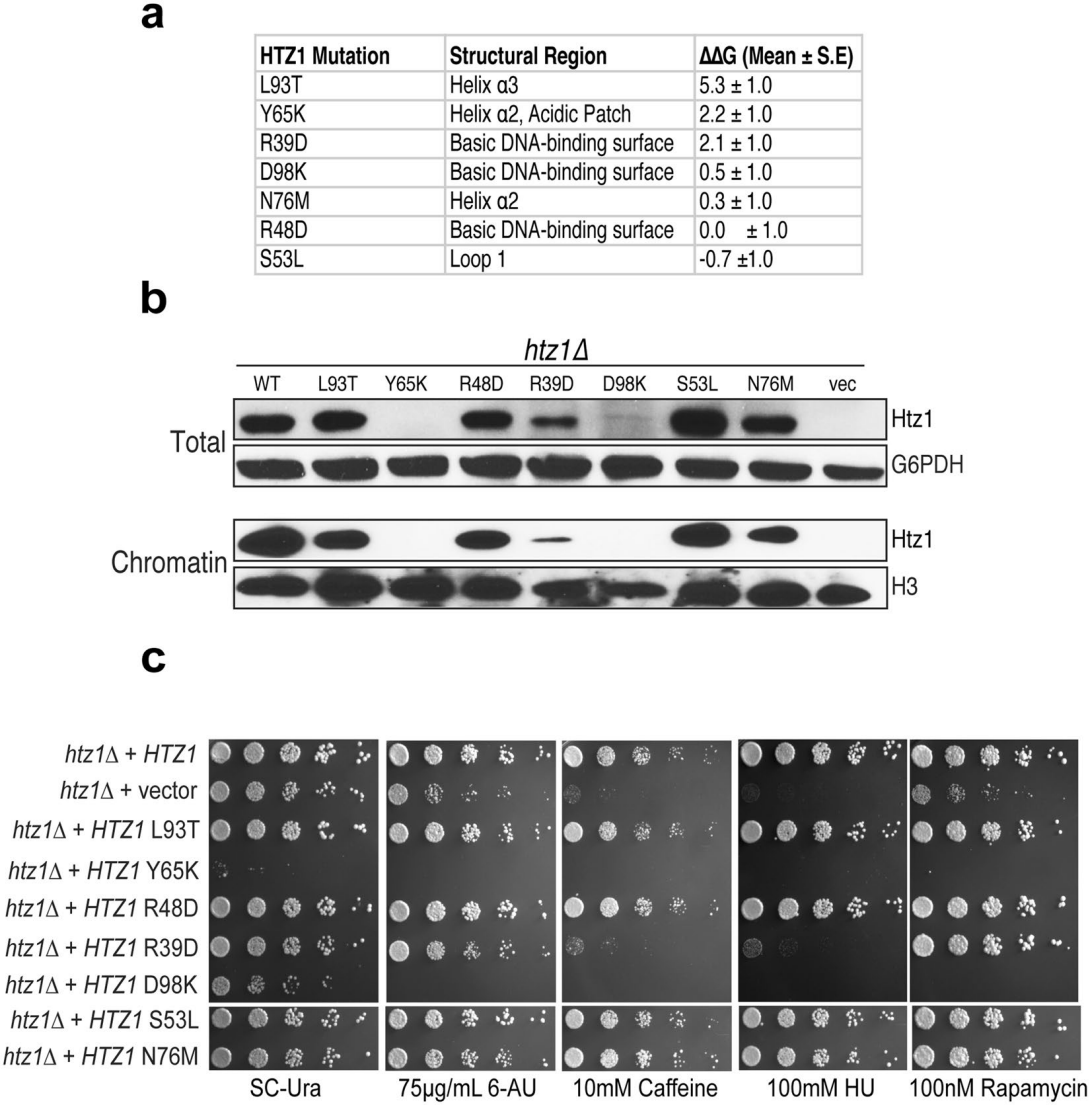
Biochemical analysis of H2A.Z mutants predicted to destabilize Chz1-H2A.Z interaction. (**a**) Table showing the list of computationally predicted mutants (HTZ1 Mutation), the region they belong to in the nucleosome structure (Structural Region), and the effect of the mutation on the computationally calculated binding energy between H2A.Z-H2B and Chz1 (ΔΔG (Mean ± S.E.)). S.E. refers to standard error of mean. (**b**) Phenotypic analyses of H2A.Z mutants predicted to destabilize H2A.Z-Chz1 interaction. Shown is are 5-fold serial dilutions of the indicated strains that were spotted on either control (Sc-Ura) or drug-containing plates. (**c**) Immunoblots showing H2A.Z levels in whole cell extracts (top) and in chromatin (bottom) from wild-type and various H2A.Z mutant strains.

To understand the cellular role of residues in H2A.Z surface that are specifically recognized by Chz1 in our DMD ensemble, we performed a computational screen (see Methods) of point mutations of H2A.Z that lie in the H2A.Z-Chz1 binding surface that we identified from DMD simulations. From this analysis, we selected three mutations in H2A.Z that were predicted to decrease its affinity towards Chz1 (Fig. 2a). These mutations were present in three different regions of Chz1-H2A.Z interface. R39 is present on the DNA binding interface of H2A.Z and the mutation R39D is predicted to destabilize Chz1 binding. As a control, we also selected R48D, in the DNA binding interface of H2A.Z, chemically similar mutation to R39D, but has no major effect on Chz1 binding. Y65 is part of the acidic patch and forms specific interactions with Chz1, with Y65K mutation predicted to severely impair Chz1 binding. As a control, we selected D98K in the acidic patch, which is predicted to have modest effect on Chz1 binding. L93, part of the helix α3, forms hydrophobic interactions with Chz1 and L93T mutation is predicted to severely destabilize H2A.Z-Chz1 interactions. S53 is part of loop1 and N76 is part of the long helix α2 and N76M and S53L are control mutations that are predicted to result in no significant change in H2A.Z-Chz1 interactions.

### H2A.Z mutants result in impairment of H2A.Z levels in chromatin

With our predicted set of mutations that result in either destabilization or no effect on Chz1-H2A.Z binding (Fig. 2a), we next determined if these mutants would affect H2A.Z function and/or incorporation into chromatin. Point mutations were generated in a wild-type (WT) *HTZ1* expression plasmid and then transformed into *htz1∆* yeast cells. We first examined the mRNA levels of the H2A.Z mutants, and found that all of them expressed H2A.Z (Supplementary Fig. 1). Given that the mRNA levels of the various point mutant were not drastically affected, we performed immunoblot analysis of the whole cell extracts (Fig. 2b, upper panels) and isolated chromatin fractions (Fig. 2b, lower panels). Our results revealed no correlation between the transcript and protein levels as the total and chromatin-bound levels of several H2A.Z mutants were significantly affected compared to WT. The most dramatic decrease in H2A.Z protein levels was observed for the Y65K and D98K mutants, followed by the R39D mutant (Fig. 2b). Next, we sought to determine if these point mutants demonstrated any biological phenotypes. To this end, we spotted the aforementioned point mutants in Fig. 2a on growth plates containing several genotoxic or transcriptional stressors as shown in Fig. 2b. Deletion of H2A.Z led to severe growth defects on plates containing 6-AU (transcription elongation inhibitor), caffeine (a pleiotropic drug that caused DNA damage), hydroxyurea (affects replication fork progression) and rapamycin (TOR inhibitor)^22^. *H2A.Z* D98K mutant revealed severe growth defects, and sensitivity to all of the genotoxic stress agents tested (Fig. 2c) similar to previous studies^12^. The *H2A.Z* Y65K mutation resulted in a very strong growth defect more severe than *H2A.Z* D98K mutant even in the absence of any stressor. To our knowledge, the finding that Y65K mutant confers severe growth defects even in the absence of genotoxic stressors is the first example of any gain-of-function mutation in H2A.Z.

### H2A.Z mutations disrupt specific chaperone interactions

**Figure 3:**
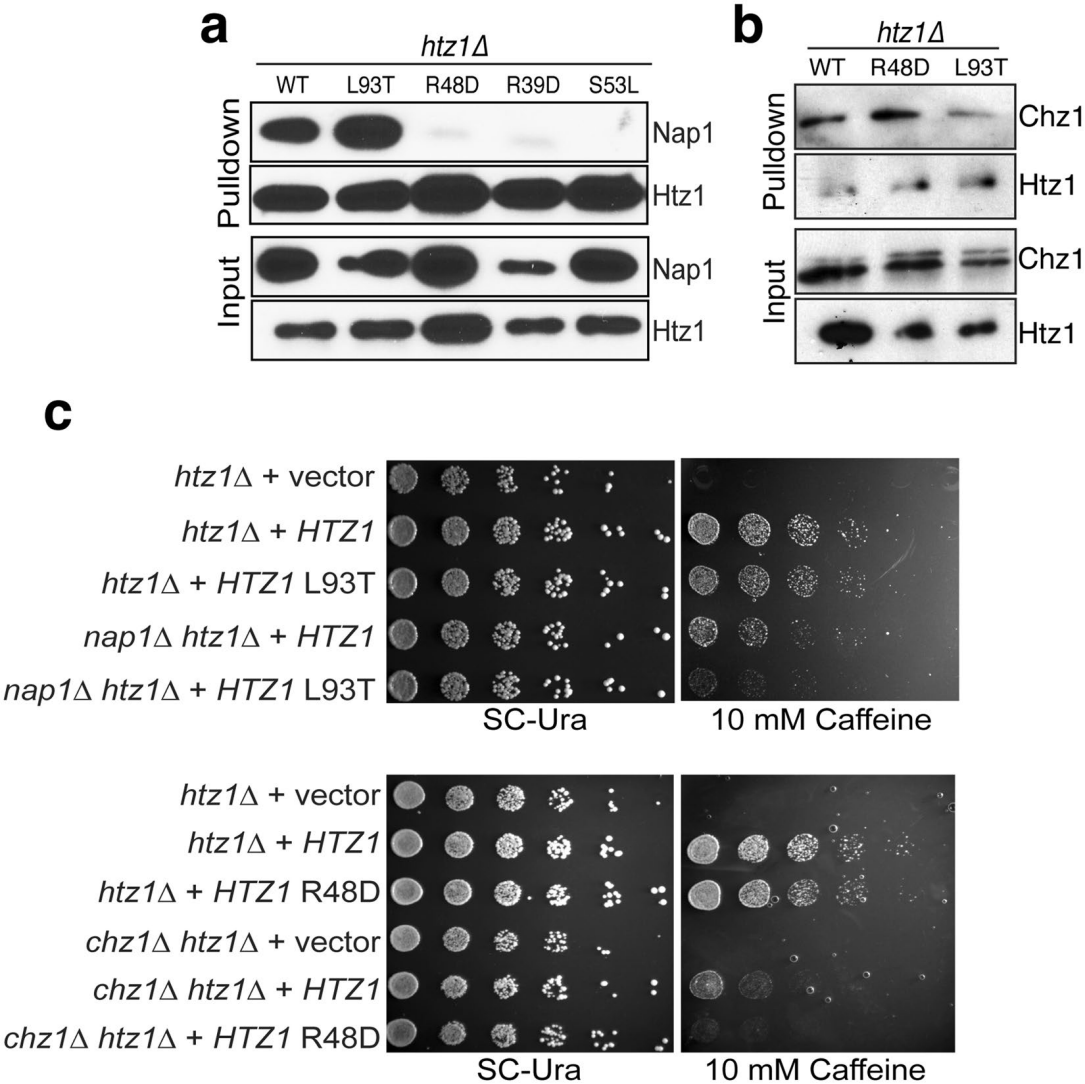
Separation-of-function mutants of H2A.Z define residues important for Nap1 and Chz1 interaction and function (**a**) Immunoblots showing the ability of various H2A.Z mutants to interact with Nap1. (**b**) Immunoblots showing the ability of various H2A.Z mutants to interact with Chz1. (**c**) 5-fold serial dilution of the indicated H2A.Z mutant strains were either plated alone or in combination with either a *CHZ1* or *NAP1* deletion on plates containing 10 mM caffeine plates.

To explore the impact of our H2A.Z mutations on Nap1 and Chz1 interaction, we utilized TAP-tagged Nap1 and Chz1 yeast strains in which *HTZ1* was deleted and transformed with the indicated *htz1* mutants. We then probed the extent to which these *htz1* mutants could co-immunoprecipitate (co-IP) either Nap1 or Chz1. The extremely low levels of the Y65K and D98K mutants precluded their use in these assays (Fig. 2b). In examination of Nap1-H2A.Z interactions, we found that the L93T mutant interacted with Nap1 at levels comparable to WT H2A.Z (Fig. 3a). Significantly, however, Nap1-H2A.Z interaction was abrogated by mutations of R48D, R39D and S53L (Fig. 3a). In examination of Chz1-H2A.Z interactions, the extent of interaction of R48D and L93T *htz1* mutants with Chz1 was altered from *H2A.Z* WT (Fig. 3b). Interestingly, significantly lower interaction was observed between the L93T mutant of H2A.Z and Chz1, which is consistent with our computational predictions that L93T would have the most severe effect on Chz1 binding (Fig. 2a). Thus, with rationally designed mutants of H2A.Z, we have uncovered two distinct classes of residues in H2A.Z that are critical for its interaction with different chaperones; R39D, R48D and S53L, part of the DNA binding interface of H2A.Z are important for Nap1 interaction, while L93T on the α3 helix is essential for Chz1 interaction. Of the mutations that we found to specifically affect either H2A.Z-Chz1 interaction (L93T) or H2A.Z-Nap1 interaction (R48D, R39D, and S53L), only the R39D mutation revealed a significant phenotype (i.e., sensitivity to caffeine and HU, similar to an *htz1∆* deletion) (Fig. 2c). The R39D sensitivity is likely explained through the fact that the chromatin levels of this mutant are significantly down (Fig. 2c), rather than a specific disruption of chaperone interaction as the R48D and S53L mutants that also disrupt Nap1-H2A.Z interaction show no phenotypes.

To understand if the mutants that lose recognition with their cognate chaperone (R39D, R48D and S53L with Nap1) show biological phenotypes in the absence of the Chz1 (mimicking a situation of loss of Nap1 and Chz1), we transformed *H2A.Z* mutants that are recognized by Nap1 into a *CHZ1* deletion strain and vice-versa. Significantly, on 10 mM caffeine plates, the L93T (a residue that causes defective recognition by Chz1) mutant showed sickness when combined with a *NAP1* deletion (Fig. 3b). Further, the R48D mutant, which prevents interaction of H2A.Z with Nap1 showed synthetic sickness when combined with a *CHZ1* deletion (Fig. 3b). These observations suggest that Nap1 and Chz1 redundantly contribute to proper SWR-C loading and deposition of H2A.Z across the genome.

### Chz1 and Nap1 are co-required for proper H2A.Z deposition

**Figure 4:**
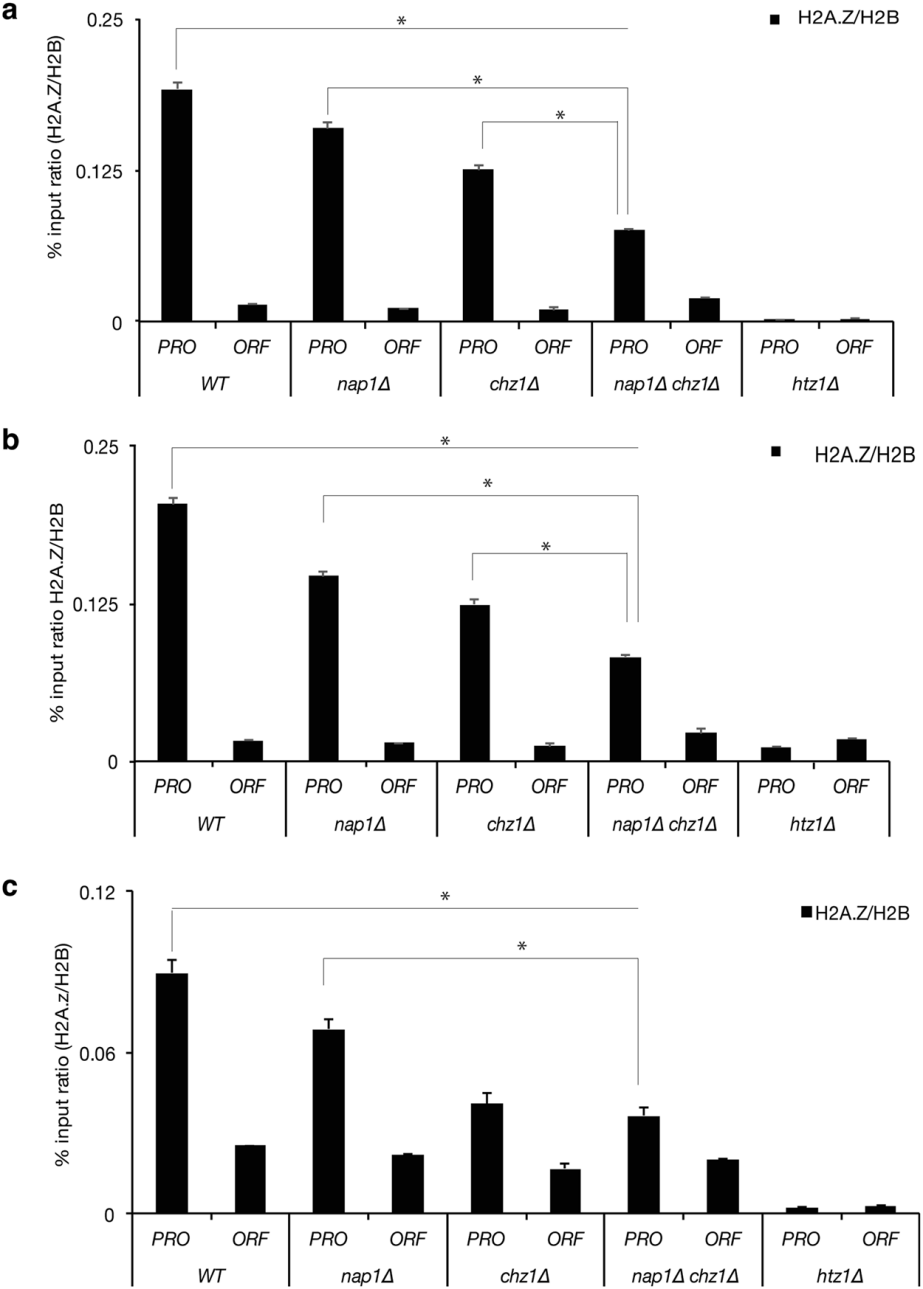
Nap1 and Chz1 function redundantly in the deposition of H2A.Z at promoters. H2A.Z levels were examined by ChIP in the indicated strains and at the promoters (PRO) and open reading frames (ORFs) of (**a**) *STE11* (**b**) *SRB5* and (**c**) *SCC2*.

Given our findings that Nap1 and Chz1 require distinct H2A.Z regions for interaction, and that mutations that block H2A.Z interaction led to several biological phenotypes, we sought to determine the impact of Nap1 and/or Chz1 loss on deposition of H2A.Z in chromatin. We utilized strains in which the genes encoding these proteins were deleted either singly or a combination (*nap1Δ*, *chz1Δ*, and *nap1Δ chz1Δ*) and then determined both the cellular and chromatin-bound levels of H2A.Z. Unexpectedly, deletion of *NAP1* and/or *CHZ1* did not result in gross changes in the overall bulk levels of H2A.Z in total and chromatin fractions in these strains (Supplementary Fig. 2). These data imply that Nap1 and Chz1 are not essential for maintaining total cellular and chromatin levels of H2A.Z. However, our observation that blocking H2A.Z–Nap1 interactions in the *chz1Δ* background (and vice-versa for H2A.Z–Chz1 interaction) results in sensitivity to caffeine suggests that Nap1 and Chz1 function might be important for locus-specific distribution of H2A.Z. Hence, we asked if Nap1 and Chz1 function is required to regulate H2A.Z levels at promoters. We performed chromatin immunoprecipitation (ChIP) followed with qPCR and examined the levels of H2A.Z at a number of gene promoters in WT and *nap1Δ*, *chz1Δ*, and *nap1Δ chz1Δ* strains. Our results showed that loss of Nap1 and Chz1 alone led to a subtle, but significantly statistic decrease (p < 0.05) in the levels of H2A.Z at promoters of *STE11, SRB5 and SCC2* (Fig. 4a–c). A double deletion of *NAP1* and *CHZ1* led to even a greater decrease in the levels of H2A.Z at the promoters of these genes, without any significant changes to the low levels of H2A.Z found in gene bodies (Fig. 4a–c). These data support the idea that Nap1 and Chz1 function in a coordinate and redundant manner in the deposition of H2A.Z at promoters.

### Chz1 and Nap1 are required to maintain heterochromatin boundaries and proper silencing

**Figure 5:**
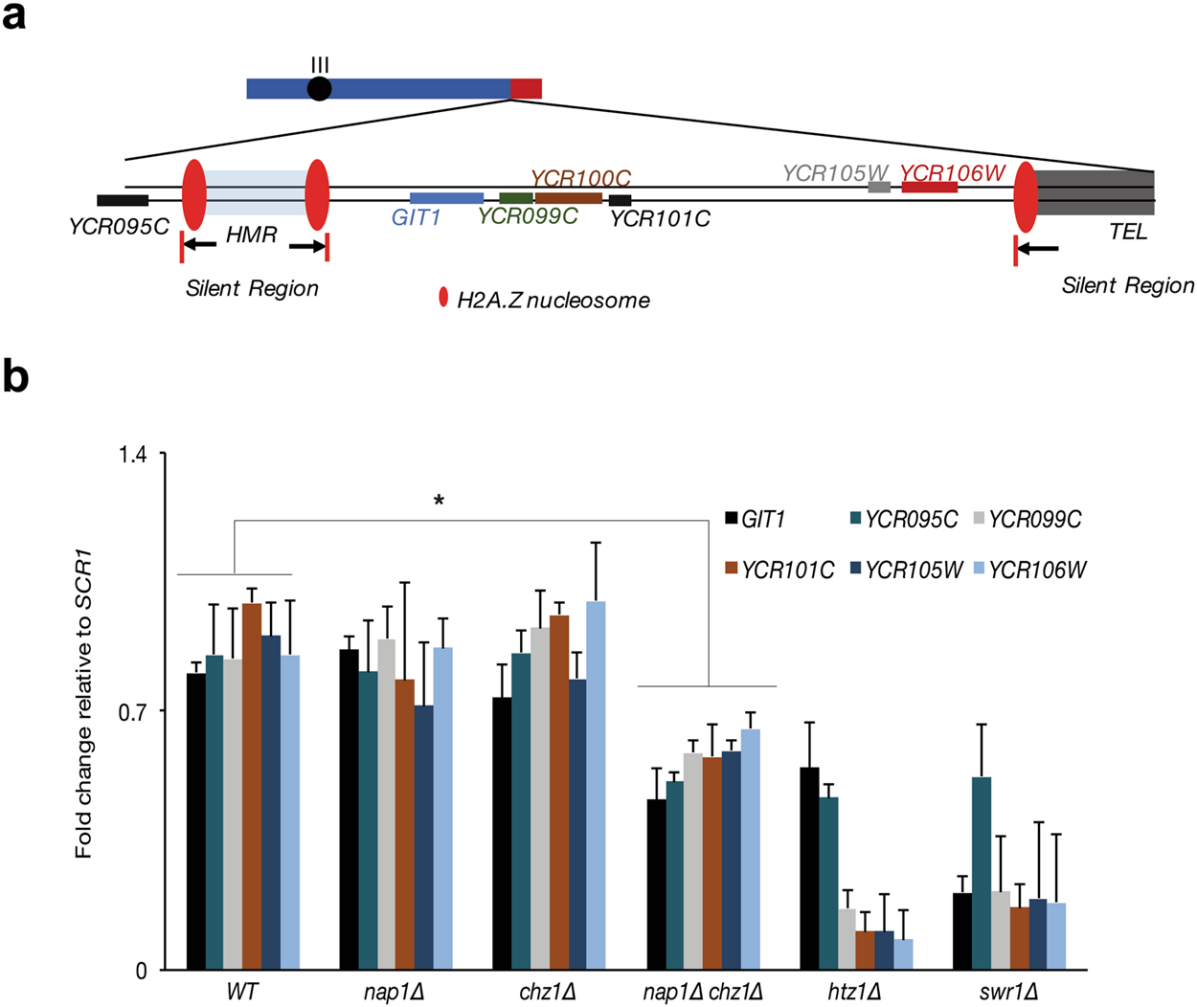
Nap1 and Chz1 function in a coordinated manner to restrict the spread of heterochromatin. (**a**) Schematic of the telomeric and mating type locus on chromosome III showing the neighboring genes. (**b**) qRT-PCR analysis of the genes represented in the schematic in different mutant strains of indicated genotypes (materials and methods). *SWR1* and *HTZ1* deletions were used a positive control. One way ANOVA was used to measure the statistical significance.

In addition to H2A.Z being localized at promoter regions, this variant is also known to be deposited at heterochromatin boundaries and to play a role in limiting heterochromatic spreading into euchromatin. We therefore asked if the loss of Nap1 and/or Chz1, in addition to their effects on H2A.Z at promoter regions, would also impact chromatin boundaries at the mating-type loci and telomeres. We adapted the system employed by Meneghini *et al*., where the spreading of the mating-type and the telomeric heterochromatin leads to silencing of genes in the neighboring regions^8^ (see schematic in Fig. 5a). We performed qRT-PCR of several genes that are adjacent to mating locus and the telomeric chromatin (Fig. 5a). Loss of Nap1 and Chz1 individually showed no significant alterations in the levels of these transcripts. In contrast, consistent with a redundant role of Nap1 and Chz1 in H2A.Z deposition, we found that loss of both Nap1 and Chz1 together led to a significant decrease in the expression of these genes – indicative of heterochromatin spreading. As positive controls, strains deleted for either *HTZ1* or *SWR*1 showed a significant decrease in these euchromatic transcripts. These results verify that Nap1 and Chz1 work in a redundant manner in the maintenance of genome organization.

## Discussion

Histone chaperones contribute to chromosome organization by regulating the deposition of canonical and variant histones at specialized locations in the genome. The budding yeast *Saccharomyces cerevisiae* contains two histone variants that are central to gene regulation and centromeric chromatin, H2A.Z and Cse4 (CENP-A), respectively. H2A.Z is deposited by the SWR-C complex, which functions with Nap1 and Chz1 by their ability to “hand-off” H2A.Z/H2B dimers to SWR-C. Yet, how these two chaperones precisely interact with H2A.Z-H2B dimers and contribute to chromatin deposition of H2A.Z is not fully understood. Here, we provide evidence that Chz1 and Nap1 function redundantly to aid SWR-C in the deposition of H2A.Z into chromatin. Our findings suggest a mechanism where Chz1 and Nap1 interact with H2A.Z-H2B dimers at unique locations on H2A.Z and contribute to SWR-C dimer loading. This model is in agreement with our findings that while the individual deletions of *NAP1* and *CHZ1* show moderate effects on H2A.Z deposition at promoters, their combined loss results in a much more severe impact on H2A.Z promoter deposition, which at heterochromatin boundaries, is manifested in a spreading of silencing into neighboring euchromatin regions.

To uncover structural underpinning of chaperone-H2A.Z interaction, we generated a refined model of H2A.Z-bound to Chz1. Structure and conformational dynamics of the Chz1-H2A.Z-H2B complex revealed a unique mode of histone recognition by Chz1 compared to many other chaperones of known structure. Importantly, the binding interface of Chz1 encompasses the DNA-binding surface of H2A.Z and the acidic patch, in addition to H2A.Z-H2B surface distal to these regions. Swc2 (VSP72) binds to the acidic patch of H2A.Z^12, 14^ and mutations in this region lead to diminished but not total abrogation of H2A.Z incorporation into chromatin^12^, implying that multiple interactions of H2A.Z (including Swc2 and Chz1) would be affected by mutations in the acidic patch. Although two acid patch mutations were generated to interrogate Chz1-H2A.Z and Swc2-H2A.Z interaction in this study (Figs 2 and 3), we found unexpectedly that expression of these mutants were slow growing and that they resulted in extremely low levels of H2A.Z at the protein level – thus precluding further binding studies (see below and Fig. 4b).

Our structural refinement strategy defined specific amino acids that were involved in recognition of the H2A.Z by Chz1 (Fig. 2a). Although our refinements were focused on the role of key H2A.Z residues involved with Chz1 interaction, we also explored the possibility these residues might also function in Nap1-H2A.Z interaction, for which no structural information is available. Fortuitously, we discovered that several mutants we designed as controls for Chz1-H2A.Z interaction were in fact specific for Nap1-H2A.Z interaction. Thus, we were able to use these mutants to delineate the functional consequences of the individual binding events by these chaperones. Future studies will be needed to better define the structure of Nap1 binding to H2A.Z-H2B, and to define the role of the residues in H2A.Z we found in Nap1-H2A.Z interface.

To better understand the role of Nap1 and Chz1 in H2A.Z deposition, we determined H2A.Z levels at specific loci across the genome after chaperone deletions. While Nap1 and Chz1 loss resulted in decrease of SWR-C-mediated decreases of H2A.Z at the promoters of genes and spreading heterochromatin, our studies also showed that loss of Nap1 and Chz1 did not lead to global decreases in bulk H2A.Z levels nor a complete loss of SWR-C-mediated deposition of H2A.Z. This was a surprising result given the clear and well-document role of Nap1 and Chz1 in nuclear shuttling of H2A.Z and H2A.Z-H2B loading onto SWR-C^4, 13^. One likely explanation for this finding is that other histone chaperones might also contribute to H2A.Z-H2B loading onto SWR-C, or that the complex itself is sufficient at binding H2A.Z-H2B dimers independently of chaperones if they are limiting or not available. It is also known that H2A.Z is deposited, albeit at low levels, broadly in chromatin independent of SWR-C, which may preclude the ability to see large decreases in H2A.Z at the bulk level in *NAP1* and *CHZ1* deletions. Regardless, it will be of significant interest to determine how H2A.Z-H2B is being loaded on SWR-C in the absence of Nap1 and Chz1, and to further explore how these chaperones contribute to H2A.Z deposition and genome function.

## Methods

### CZB constructs used in structural studies

We used the NMR structure of CZB (PDB ID: 2JSS) as a starting structure for our simulations. This construct consists of H2A.Z (residues 22–118, UniProt ID: Q12692), H2B (residues 36–130, UniProt ID: P02294) and Chz1 (63–124). The NMR sample was constructed by connecting the C-terminus of H2B and N-terminus of H2A.Z to form a single peptide chain. In the construct used in our simulations, we treated H2A.Z and H2B as separate chains.

### CZB DMD simulations

We employed replica exchange, parallelized discrete molecular dynamics to sample ensemble of conformations of the CZB complex. The DMD simulation methodology is described in detail elsewhere^23–25^. Briefly, we use Medusa force field^26^ that is based on CHARMM19 non-bonded potentials^27^, EEF1 implicit solvation parameters^28^, geometry-based hydrogen bond potential and long-range electrostatic potential^29^ to model various macromolecular interactions. The time unit of the all-atom DMD simulations is ~50 femtoseconds (fs) and the temperature is maintained using Anderson’s thermostat^30^. We performed ten sets of DMD simulations for ~1 x 10^6^ time units with a total of 16 replicas, resulting in total sampling of ~160 x 10^6^ time units, or ~8 μs. The 16 replicas were set at following temperatures: 0.480, 0.495, 0.512, 0.528, 0.546, 0.563, 0.581, 0.600, 0.619, 0.638, 0.658, 0.679, 0.700, 0.722, 0.744 and 0.767 ϵ (reduced units^25^); roughly corresponds to 240–383K). To increase sampling of Chz1, so as to optimize the Chz1-histone interface, while biasing the simulations towards known experimental data, we utilized two sets of constraints: (i) the backbone atoms of H2A.Z (Residues: 26–113) and H2B (40–129) were harmonically constrained to their starting coordinates with a spring constant of 0.4 kcal.mol^−1^.atom^−1^ (ii) the distance between a subset of atoms of Chz1 and the histones (determined using Nuclear Overhauser Effect (NOE) – NMR spectroscopy), were restrained with a two-well potential (Supplementary Figure 3). The list of NOE restraints were utilized from earlier study with minor modifications^15^: (i) Since our force field does not consider aliphatic hydrogens, the constraints containing aliphatic hydrogens were modified to contain the carbon atom to which the hydrogens were bonded, and the constraint length was increased by 1 Å to reflect the additional bond-length; (ii) all restraints were increased by 1 Å to account for the standard deviation of the NOE signal.

### Simulations analysis – identifying a refined CZB ensemble

We calculated several parameters of the simulation snapshots to identify a subset of snapshots (the refined ensemble) that features an optimized protein-protein interface and also agrees well with experimental distance restraints. We used our published electrostatic potential^24^, Medusa potential^26^ and the mean NMR violations as criteria to identify the refined ensemble. If the distance between two atoms is higher than the experimental distance, the difference between the observed distance and the experimental distance gives the violation value for that distance restraint. The average of such values over all distance restraints gives the mean NMR violation. After filtering out high energy structures and structures with high NMR violations, we identified a refined ensemble of 1454 structures. We determined the mean NMR violation of this refined ensemble as follows. Since the NOE signal is proportional to the ensemble-averaged 1/r^6^ between two atoms, we determined the average 1/r^6^ from our filtered ensemble and then calculated r from that average. This distance r for each distance restraint was used to calculate the mean NMR violation of the ensemble. We observe the mean NMR violation of this ensemble to be lower than observed for the published NMR ensemble, indicating excellent agreement with experimental structural data for the CZB complex.

### Interface analysis

We define a contact as a pair of atoms (one from H2A.Z-H2B and the other from Chz1) that are not hydrogen and are within a distance of 6 Å of each other. For the refined ensemble from DMD, we calculated the average number of contacts formed by each residue of H2A.Z-H2B with Chz1. We colored each residue in the H2A.Z-H2B interface with Chz1 (on structural figures) based on the average number of contacts formed by the residue.

### Estimation of change in binding affinity upon mutation

We calculated the change in binding energy of H2A.Z-H2B to Chz1 upon mutation using *Eris* protocol of Medusa^31–33^. We performed 17 possible point mutations (all residues except proline, cysteine and the native amino acid) at all residues of H2A.Z that were part of the interface with Chz1 (Fig. 1c) to determine a list of mutations that destabilize the CZB complex. Medusa calculations involve a Monte Carlo based simulated annealing procedure that uses rotamer libraries of amino acids for fast minimization of its energy function while leaving the backbone fixed. Medusa uses a combination physics-based terms (van der Waals, hydrogen bond, solvation) and knowledge-based terms (backbone and side chain torsions). We averaged the free energy obtained from at least 500 Medusa calculations for each of the six centroid structures (from the refined ensemble) to obtain ∆∆G for each mutation. We define ∆∆G as:

$${\rm{\Delta }}{\rm{\Delta }}{\rm{G}}=({{\rm{\Delta }}{\rm{G}}}_{\mathrm{Complex} \mbox{-} \mathrm{Mut}}-{{\rm{\Delta }}{\rm{G}}}_{{\rm{H}}2{\rm{A}}.{\rm{Z}}-{\rm{H}}2{\rm{B}}-{\rm{Mut}}})-({{\rm{\Delta }}{\rm{G}}}_{\mathrm{Complex}-\mathrm{Mut}={\rm{WT}}}-{{\rm{\Delta }}{\rm{G}}}_{{\rm{H}}2{\rm{A}}.{\rm{Z}}-{\rm{H}}2{\rm{B}}-{\rm{WT}}}),$$

where ΔG_Complex-Mut_ is the stability of the mutant H2A.Z-H2B-Chz1 complex, ΔG_H2A.Z-H2B-Mut_ is the stability of the mutant H2A.Z-H2B dimer, ΔG_Complex-WT_ is the stability of the wild type H2A.Z-H2B-Chz1 complex and ΔG_H2A.Z-H2B-WT_ is the stability of the wild type H2A.Z-H2B dimer. Thus, a destabilizing mutation would result in a positive ∆∆G.

### Yeast strains and plasmids

The chromosomal copy of the histone H2A.Z (*HTZ1*) gene was deleted in the following yeast strains: (1) a strain bearing TAP-tagged Chz1 (Open Biosystems), (2) a strain bearing TAP-tagged Nap1 (Open Biosystems), (3) a strain bearing *nap1Δ* (Open Biosystems), (4) and a strain bearing *swr1Δ* (Open Biosystems). *HTZ1* was replaced with the NatMX gene deletion cassette in these strains using PCR mediated gene disruption^34^. The resulting yeast strains were screened on media containing cloNAT (Werner Bioagents) at 100 μg/mL. Strains were confirmed by PCR amplification and Western blot analysis. Plasmids containing either a WT or mutant H2A.Z allele (see below) were transformed into the resulting deletion strains. Yeast strains used in this study are listed in Supplementary Table 1.

### Plasmid construction

The pRS416-based H2A.Z-2Flag vector (a gift from Dr. C. Wu) was used as a template to generate all subsequent plasmids bearing mutations to H2A.Z. All mutations were made by site directed mutagenesis (QuikChange, Stratagene) and confirmed by sequencing.

### Whole cell extract, Chromatin fractionation, and Immunoblotting

For analysis of total protein levels, whole cell extracts were made using established protocols^35^. Chromatin was isolated from strains using established protocols^36^. Electrophoresis and immunoblot analysis were performed as described elsewhere^37^.

### Co-immunoprecipitation

*HTZ1* was deleted in the TAP-*CHZ1* and TAP-*NAP1* (from Open Biosystems) and co-immunoprecipitation was performed as before^10^.

### Chromatin-immunoprecipitation

ChIP was performed as described previously using the H2A.Z antibody (Active Motif Cat #39648). Data are represented as percent input and the primer sequences of the ORFs used in the text will be available upon request.

### Quantitative real-time PCR

RNA was extracted form asynchronously growing log phase cells of the indicated strains. cDNA was synthesized using the First Strand cDNA synthesis system and the was subjected to qRT-PCR using SYBR green (Bio-Rad). Relative quantities of the transcripts was measured using the ΔΔCt method. Data are represented as mean over standard deviation of three independent experiments.

## Electronic supplementary material

Supplementary Information
