# Supplementary material for: Redundant Functions for Nap1 and Chz1 in H2A.Z Deposition
Source: Sci Rep. 2017 Sep 7;7:10791. doi: 10.1038/s41598-017-11003-8 (PMC5589762; doi:10.1038/s41598-017-11003-8)
Supplement: Supplementary file 1 — Supplementary Information [file 41598_2017_11003_MOESM1_ESM.pdf]

Supplementary Information

Redundant Functions for Nap1 and Chz1 in H2A.Z Deposition

Raghuvar Dronamraju[§1], Srinivas Ramachandran[§1,2], Deepak K. Jha[§1,3], Alexander T. Adams[1], Julia V. DiFiore[4], Michael A. Parra[5], Nikolay V. Dokholyan*[1,5] & Brian D. Strahl*[1,4,6]

[1]Department of Biochemistry and Biophysics, University of North Carolina at Chapel Hill, Chapel Hill, NC 27599 USA, [4]Curriculum in Genetics and Molecular Biology, University of North Carolina, Chapel Hill, NC 27599, [6]Program in Molecular and Cellular Biophysics, University of North Carolina at Chapel Hill, Chapel Hill, NC 27599 USA, [6]Lineberger Comprehensive Cancer Center, University of North Carolina at Chapel Hill, Chapel Hill, NC 27599 USA

[2]Current Address: Division of Basic Sciences, Fred Hutchinson Cancer Research Center, Seattle, WA 98109 USA, [3]Current Address: Division of Hematology/Oncology, Department of Medicine, Children's Hospital Boston, Boston, MA.

[5]Current Address: Department Susquehanna University, Selinsgrove, PA, 17870 USA

[§]These authors contributed equally

*Corresponding Authors: e-mail: brian_strahl@med.unc.edu e-mail: dokh@unc.edu

Supplementary information comprises:

Supplementary Figures S1

Supplementary Figures S2

Supplementary Figures S3

Supplementary Table 1

Supplementary Figure 1. H2A.Z (Htz1) mutants do not show defective transcript levels.

RT-PCR of mRNA isolated from various H2A.Z point mutants show that the point mutants show almost wild-type levels of H2A.Z transcripts. Act1 acts as the loading control.

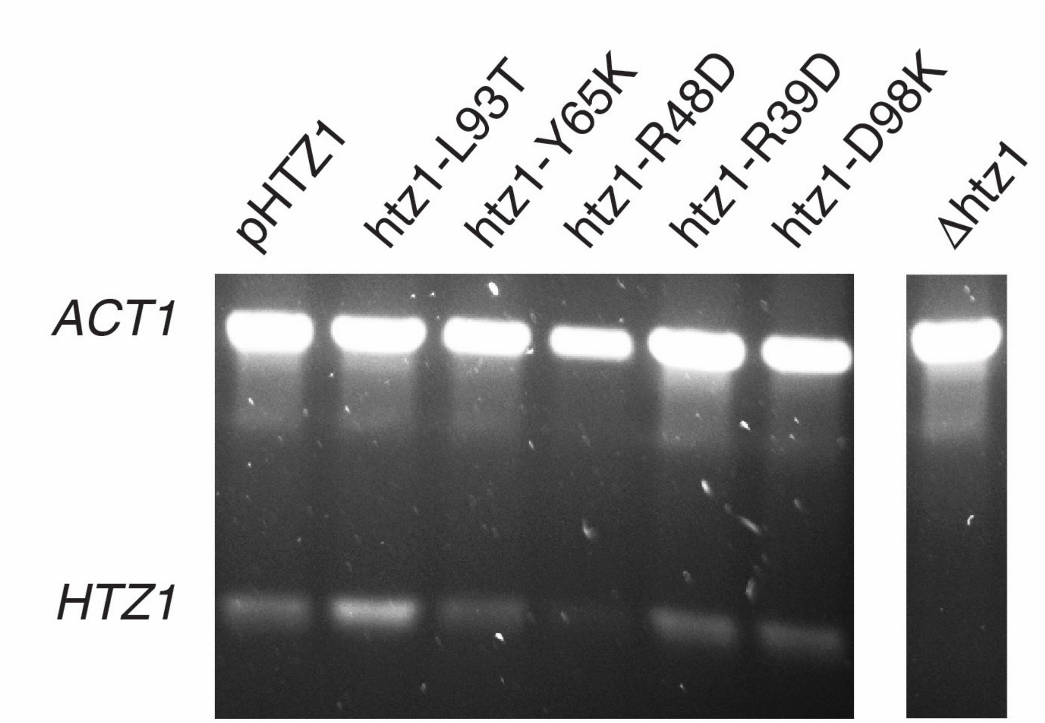

Supplementary Figure 2. H2A.Z (Htz1) levels in single and double mutants of Nap1 and Chz1. (a) Chromatin association of H2A.Z for the indicated strains as described in materials and methods. (b) Relative quantitation of H2A.Z levels on chromatin as shown from the immunoblot in a.

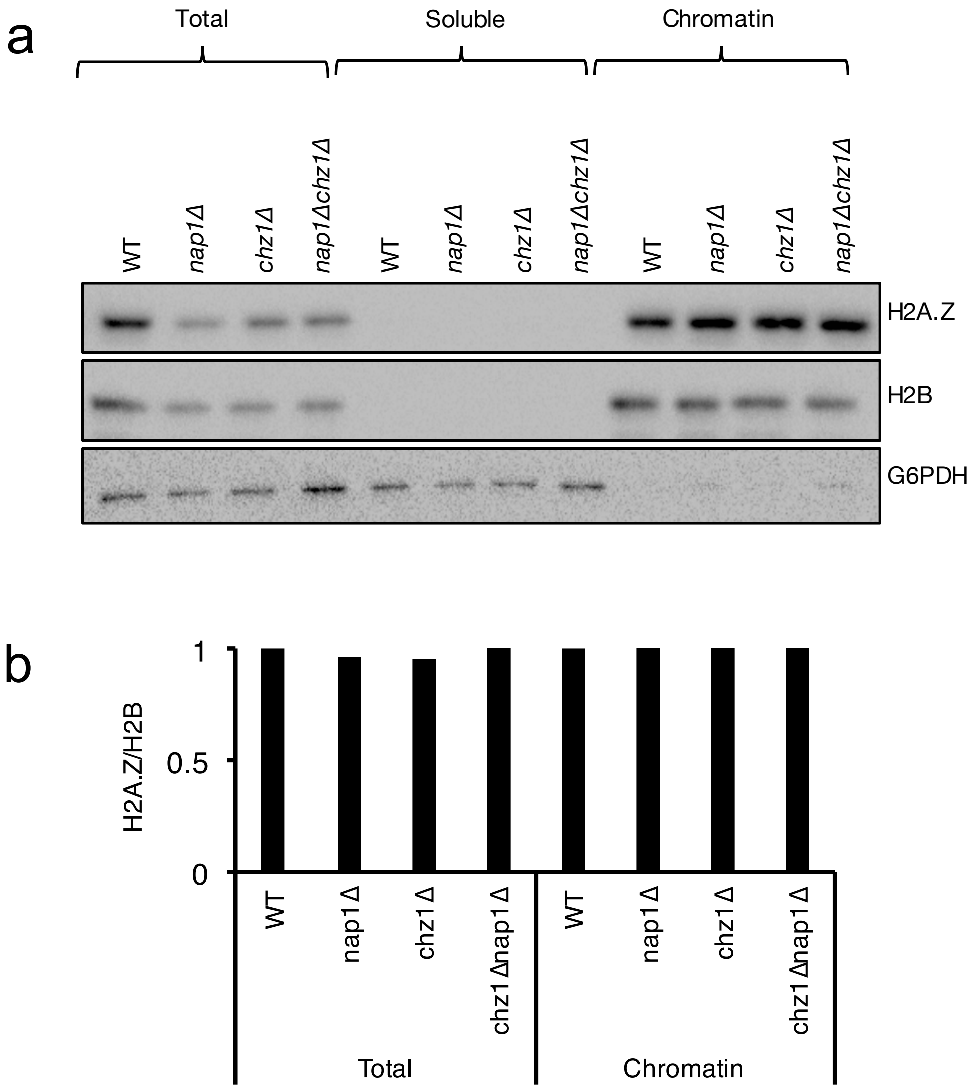

Supplementary Figure 3. Pairwise potential used in DMD to impose NMR constraints.

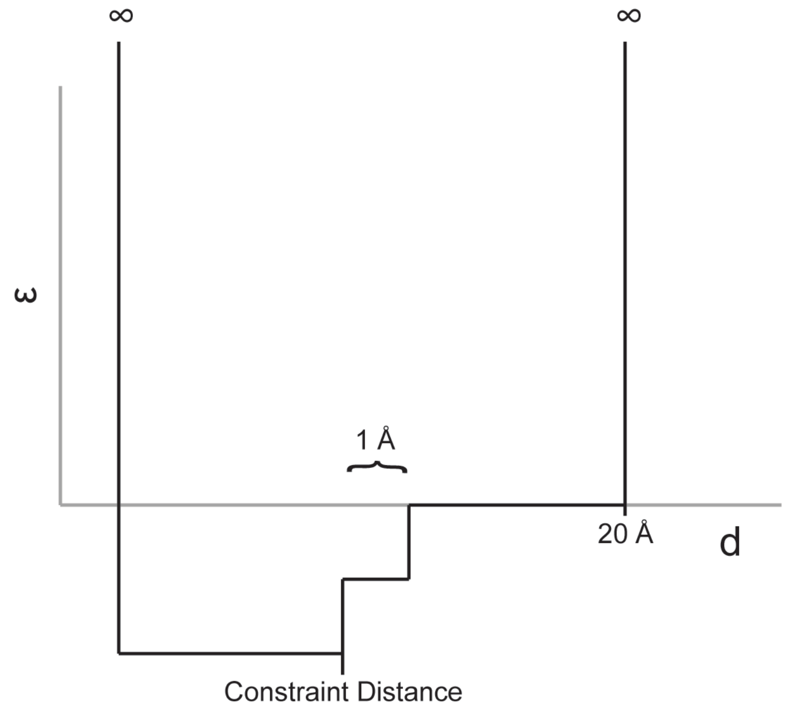

Supplementary Table 1: List of yeast strains and genotypes used in these studies

Strain	Experiment	Genotype	Source
BY4741	Endogenous H2A.Z expression levels	*MATa (his3-Δ1 leu2-Δ0 met15-Δ0 ura3-Δ0)*	Open Biosystems
YMP050	*htz1Δ* in BY4741 background	*MATa (his3-Δ1 leu2-Δ0 met15-Δ0 ura3-Δ0; htz1Δ::KanMX4)*	Open Biosystems
YMP295	*nap1Δ* in BY4741 background	*MATa (his3-Δ1 leu2-Δ0 met15-Δ0 ura3-Δ0; nap1Δ::KanMX4)*	Open Biosystems
YMP211	*chz1Δ* in BY4741 background	*MATa (his3-Δ1 leu2-Δ0 met15-Δ0 ura3-Δ0; chz1Δ::KanMX4)*	Open Biosystems
RDY420	*nap1Δ nap1Δ* in BY4741 background	*MATa (his3-Δ1 leu2-Δ0 met15-Δ0 ura3-Δ0; nap1Δ::KanMX4 chz1Δ::natNT2)*	This study
YMP179	*swr1Δ* in BY4741 background	*MATa (his3-Δ1 leu2-Δ0 met15-Δ0 ura3-Δ0; swr1Δ::KanMX4)*	Open Biosystems
YMP213	*htz1Δ* in *CHZ1*-TAP tagged background (Open Biosystems)	*MATa his3-Δ1 leu2-Δ0 met15-Δ0 ura3-Δ0 CHZ1-TAP htz1Δ::kanMX4*	This study
YMP216	*htz1Δ* in *NAP1*-TAP tagged background (Open Biosystems)	*MATa his3-Δ1 leu2-Δ0 met15-Δ0 ura3-Δ0 NAP1-TAP htz1Δ::kanMX4*	This study
YMP065	*htz1Δ* in BY4741 background covered with plasmid pMP008	Isogenic to YMP050, plus pMP008 (*CEN6 URA HTZ1*)	This study
YMP286	*HTZ1* L93T in h2a.zΔ background	Isogenic to YMP050, plus pMP115 (*CEN6 URA HTZ1 L93T*)	This study
YMP287	*HTZ1* Y65K in h2a.zΔ background	Isogenic to YMP050, plus pMP116 (*CEN6 URA HTZ1Y65K*)	This study
YMP288	*HTZ1* R48D in h2a.zΔ background	Isogenic to YMP050, plus pMP117 (*CEN6 URA HTZ1 R48D*)	This study
YMP289	*HTZ1* R39D in h2a.zΔ background	Isogenic to YMP050, plus pMP119 (*CEN6 URA HTZ1 R39D*)	This study
YMP290	*HTZ1* D98K in h2a.zΔ background	Isogenic to YMP050, plus pMP120 (*CEN6 URA HTZ1 D98K*)	This study

YMP291	*HTZ1* S53L in h2a.zΔ background	Isogenic to YMP050, plus pMP121 (*CEN6 URA HTZ1 S53L*)	This study
YMP292	*HTZ1* N76Min h2a.zΔ background	Isogenic to YMP050, plus pMP122 *(CEN6 URA HTZ1 N76M*)	This study
YMP300	*nap1Δ* and *htz1Δ* double deletion	*MATa (his3-Δ1 leu2-Δ0 met15-Δ0 ura3-Δ0; nap1Δ::KanMX4 htz1Δ::NatMX)*	This study
YMP301	*chz1Δ* and *htz1Δ* double deletion	*MATa (his3-Δ1 leu2-Δ0 met15-Δ0 ura3-Δ0; swc2Δ::KanMX4 htz1Δ::NatMX)*	This study
YMP307	Wild-type control for TAP-Tagged pulldown of Chz1	Isogenic to YMP213, plus pMP008 (*CEN6 URA H2A.Z*)	This study
YMP308	*HTZ1* L93T for TAP-Tagged pulldown of Chz1	Isogenic to YMP213, plus pMP115 (*CEN6 URA HTZ1 L93T*)	This study
YMP310	*HTZ1* R48D for TAP-Tagged pulldown of Chz1	Isogenic to YMP213, plus pMP117 (*CEN6 URA HTZ1 R48D*)	This study
YMP311	*HTZ1* R39D for TAP-Tagged pulldown of Chz1	Isogenic to YMP213, plus pMP119 (*CEN6 URA HTZ1 R39D*)	This study
YMP313	*HTZ1* S53L for TAP-Tagged pulldown of Chz1	Isogenic to YMP213, plus pMP121 (*CEN6 URA HTZ1 S53L*)	This study
YMP316	Wild-type control for TAP-Tagged pulldown of Nap1	Isogenic to YMP216, plus pMP008 (*CEN6 URA H2A.Z*)	This study
YMP317	*HTZ1* L93T for TAP-Tagged pulldown of Nap1	Isogenic to YMP216, plus pMP115 (*CEN6 URA HTZ1 L93T*)	This study
YMP319	*HTZ1* R48D for TAP-Tagged pulldown of Nap1	Isogenic to YMP216, plus pMP117 (*CEN6 URA HTZ1 R48D*)	This study
YMP320	*HTZ1* R39D for TAP-Tagged pulldown of Nap1	Isogenic to YMP216, plus pMP119 (*CEN6 URA HTZ1 R39D*)	This study
YMP322	*HTZ1* S53L for TAP-Tagged pulldown of Nap1	Isogenic to YMP216, plus pMP121 (*CEN6 URA HTZ1 S53L*)	This study
YMP323	*HTZ1* N76M for TAP-Tagged pulldown of Nap1	Isogenic to YMP216, plus pMP122 *(CEN6 URA HTZ1 N76M*)	This study
